# Cultural Beliefs and Perceptions of Breast Cancer Among Adolescent Girls in Semi‐Urban Bangladesh: A School‐Based Cross‐Sectional Study

**DOI:** 10.1002/puh2.70369

**Published:** 2026-09-15

**Authors:** Ryadul Alam, Sadia Akter, Tanaa Mohammad, Rowshanara Rini, Mst. Mariyam, Afrana Akter Priti, Aishwaria Mitra Ayshee, Md. Shahriar Hossen Sayem, Afsana Mohammad Mimi, Zubaida Iftekhar, Md. Habibullah Talukder, Md. Tajuddin Sikder

**Affiliations:** ^1^ Department of Public Health & Informatics Jahangirnagar University Dhaka Bangladesh; ^2^ St Francis College Brooklyn New York USA; ^3^ National Institute of Cancer Research & Hospital Dhaka Bangladesh

**Keywords:** adolescent girls, Bangladesh, breast cancer, cultural belief

## Abstract

**Background:**

Breast cancer is the most common cancer among women and poses a growing public health burden in developing countries, including Bangladesh. Cultural stigma, limited awareness, and delayed recognition contribute to late diagnosis, particularly among younger females. This study examines cultural beliefs and perceptions of breast cancer among adolescent girls in semi‐urban Bangladesh.

**Methods:**

A school‐based cross‐sectional study was conducted among adolescent girls in semi‐urban Savar, Dhaka, Bangladesh, from January to May 2025. Four schools were randomly selected, and 384 participants were recruited using stratified random sampling. Cultural beliefs about breast cancer were assessed using a modified 13‐item Ferrans Cultural Beliefs Scale, translated into Bangla using a committee‐based approach. Data were analyzed using SPSS version 26 with descriptive statistics, *t*‐tests, one‐way ANOVA, and multiple linear regression.

**Result:**

Cultural belief score is 7.38 ± 2.08, rooted misconceptions among participants. Multiple linear regression analysis showed that a higher class of study was positively associated with cultural belief scores (*β* = 0.236, *p* = 0.037), whereas having a family history of breast cancer was strongly associated with lower cultural belief scores (*β* = −1.979, *p* < 0.001). Reliance on friends as a source of information significantly increased cultural belief scores (*β* = 1.358, *p* < 0.001). Parental education and average monthly family income were not significant predictors (*p* > 0.05).

**Conclusion:**

Adolescent girls in semi‐urban Bangladesh showed a moderate level of cultural beliefs about breast cancer, with a higher class of study, reliance on friends as an information source, and the absence of a family history of breast cancer associated with a greater endorsement of these beliefs, underscoring the need for early, culturally sensitive school‐based education.

## Graphical abstract

In a school‐based study of 384 adolescent girls in semi‐urban Bangladesh, cultural beliefs regarding breast cancer were moderate overall. Higher class of study and peer information sources were linked to stronger beliefs, whereas family history was linked to fewer misconceptions, underscoring the need for school‐based education.

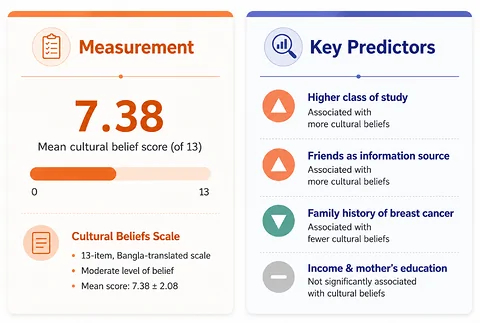

## 1 Introduction

Cancer is a major public health concern globally, with an estimated 20 million new cases and 9.7 million deaths globally. Although lung cancer is the most common cancer in men (12.4% of all new cases), breast cancer is by far the most common cancer among women, accounting for 11.6% of all new cancer cases [1]. More than half of the breast cancer deaths occur in economically developing countries. The South Asian countries are experiencing an epidemic of breast cancer in secrecy. About 588 million women above the age of 15 are dealing with a growing breast cancer epidemic [2].

In Bangladesh, breast cancer represents a public health burden and ranks second only to cervical cancer among women. Together, these two cancers account for nearly 38% of all female cancers nationwide. The incidence rate of breast cancer in Bangladesh is estimated at 22.5 per 100,000 women, with a mean age of diagnosis of 41.8 years, indicating a substantial burden among premenopausal women [3, 4]. Despite this, the awareness and timely recognition of breast cancer remain low, particularly among younger females [5]. Cultural taboos surrounding the female body, modesty norms, fear of social judgment, and reluctance to discuss breast‐related health issues contribute significantly to delayed health‐seeking behavior [6].

Most of the breast cancer cases are diagnosed at advanced stages in Bangladesh [7]. This pattern is closely linked to sociocultural barriers, including misconceptions about cancer, stigma associated with the disease, fear of discrimination, and limited open communication within families and communities. In semi‐urban areas, these challenges are further compounded by inadequate health education, limited access to accurate information, and persistent traditional beliefs that frame cancer as a fatal or shameful condition rather than a treatable disease [8, 9]. Distance to healthcare facilities, lack of time, inconvenient service hours, and lack of health insurance or awareness about screening centers are significant obstacles [10].

Cultural beliefs about illness are broadly understood as the shared understandings, values, and assumptions that shape how a community interprets the cause, meaning, and appropriate response to a health condition [11]. In the context of breast cancer specifically, cultural beliefs have been shown to function as a distinct determinant of screening and help‐seeking behavior, separate from factual knowledge deficits alone. For example, a study among rural Vietnamese women found that erroneous beliefs about breast lumps, self‐help remedies, faith‐based protection, and the perceived futility of treatment were common even where general cancer awareness existed, and that these beliefs were more pronounced among younger and lower income women [12]. Individuals with identical factual knowledge about breast cancer may still differ substantially in whether they consider the disease shameful, treatable, or worth discussing openly, and these evaluative and normative perceptions directly shape screening intention and delay [11].

Adolescence is a critical developmental period during which such health beliefs, attitudes, and perceptions are formed [13]. Cultural beliefs acquired during this stage often shape lifelong health behaviors and influence future decision‐making related to disease prevention and healthcare utilization. Cultural beliefs, stigma, and lack of family or community support can discourage screening, especially in more conservative or close‐knit communities [14]. The most consistently reported barrier is insufficient knowledge about breast cancer and available screening methods; many young women are unaware of the importance of screening, how to perform self‐exams, or where to access services [15, 16, 17]. Fear of pain, fear of a cancer diagnosis, embarrassment, and anxiety about the screening procedure or results deter many young women from participating [14].

Adolescents form health beliefs through a distinct set of channels compared with adults, primarily media, peers, and family rather than through structured clinical education, and this informal pathway can just as easily transmit misconceptions as accurate information [18]. Peer norms exert a strong influence on how adolescents interpret and respond to health‐related topics, including those considered sensitive or taboo within the family [19]. In settings where breast cancer is rarely discussed openly at home, these informal, peer‐ and media‐driven channels may become the dominant source through which adolescent girls construct their understanding of the disease.

However, in Bangladesh, adolescent girls, particularly those attending high schools in semi‐urban settings, remain an understudied population in breast cancer research. Existing studies have largely focused on adult women or university students, overlooking the early formation of beliefs, misconceptions, and stigma among school‐aged girls. Understanding cultural beliefs about breast cancer among adolescent girls is especially important in semi‐urban contexts, where traditional values coexist with increasing exposure to modern education and media. These settings represent a transitional space in which cultural norms, gender expectations, and health knowledge interact in complex ways.

Therefore, this study aims to assess cultural beliefs and perceptions about breast cancer and to identify their sociodemographic and informational predictors among female high school students in semi‐urban areas of Bangladesh. By focusing on adolescent girls within a school‐based setting and extending prior knowledge‐focused work in this population [20], this research seeks to address a critical gap in the literature and generate evidence to inform culturally appropriate awareness programs and early preventive strategies tailored to this vulnerable population.

## 2 Methodology

### 2.1 Study Design and Participants and Sampling

This school‐based cross‐sectional study was conducted among adolescent girls (aged 13–19 years) in a semi‐urban area of Dhaka, Bangladesh. The study was carried out over a 4‐month period, from January 10, 2025 to May 10, 2025, across four government schools that were selected randomly from Savar Upazila, Dhaka District (semi‐urban area). The survey was administered in classroom settings during school hours with the permission of the authorities, and each participant required approximately 15–20 min to complete the questionnaire.

### 2.2 Sample Size

The minimum sample size was calculated using Yamane's simplified formula for finite populations:

$$n=\frac{N}{1+N\left(e^{2}\right)}$$

$$n=\frac{9400}{9400{\left(0.05\right)}^{2}}$$

=383.7≈384

where *n* is the required sample size, *N* is the total eligible population (9400; total number of eligible female students in the 4 selected schools), and *e* is the precision level (0.05). Using this approach, the minimum estimated sample size was **384** participants. A precision level of *e* = 0.05 was adopted, consistent with conventional practice for school‐based cross‐sectional health surveys that was used in previous institutional Bangladeshi study [20, 21]. Yamane's formula assumes simple random sampling; because the present study instead used a proportionate, two‐stage stratified design with strata (schools) that were broadly homogeneous with respect to the eligibility criteria, no additional design–effect inflation was applied to the calculated sample size.

### 2.3 Sampling Technique

Two‐stage sampling procedures were used, with each school considered a stratum. The number of participants selected from each school was proportional to the total number of eligible students enrolled, ensuring a proportional representation across the study population.

#### 2.3.1 Stage 1 (School Selection)

Four schools in Savar were selected using simple random selection from the list of eligible schools from the existing government secondary school and college list (excluding only boys’ school or college) [22].

#### 2.3.2 Stage 2 (Student Selection)

A proportionate stratified random sampling technique was used, with each school considered a stratum. The number of participants selected from each school was proportional to the total number of eligible students enrolled, ensuring balanced representation across the study population.

The sample size of 384 was proportionately allocated across the four selected schools using the formula:

$$n_{i}=\left(N_{i}\;/\;N\right)\times n$$

where *N_i_* is the number of eligible female students (Classes 9–12) enrolled in each school, as obtained from the respective school administrative registries at the time of data collection; *N* is the total eligible population across the four schools (9400); and *n* is the final calculated sample size (384) (Table 1).

**TABLE 1:** Proportionate allocation of study sample across the selected schools in Savar, on the basis of eligible female student population (Classes 9–12).

School	Eligible population (*N_i_*)	Sampling fraction (*N_i_*/*N*) (%)	Sample size (*n_i_*)	% of total sample
School A	2790	29.7	114	29.7
School B	2399	25.5	98	25.5
School C	2105	22.4	86	22.4
School D	2105	22.4	86	22.4
Total	*N* = 9400	100	384	100

### 2.4 Inclusion and Exclusion Criteria

Inclusion criteria were (i) female students (Classes 9–12) who were enrolled in the selected schools and (ii) willingness to participate in the survey.

Exclusion criteria included participants who provided incomplete responses.

### 2.5 Study Instruments

#### 2.5.1 Independent Variable

Sociodemographic and other variables, including age, class of study, academic section, marital status, religion, parental education, average monthly family income, family history of breast cancer, knowledge of breast self‐examination (BSE), and source of breast‐health information, were also collected via the same self‐administered questionnaire. These variables were included as covariates commonly examined in the cultural‐beliefs and adolescent health‐behavior literature, to test for potential sociodemographic and informational correlates of cultural beliefs about breast cancer [12, 20].

#### 2.5.2 Dependent Variable

A modified version of the Cultural Beliefs Scale that was first created by Ferrans was used to evaluate cultural beliefs about breast cancer. This scale was formulated to assess the degree to which beliefs that are culturally based serve to act as impediment to early breast cancer diagnosis, and that it may also be a contributor to the late diagnosis.

The initial scale has 17 questions divided into four conceptual areas that are beliefs about breast lumps (4 items), self‐help techniques (4 items), faith‐based beliefs (4 items), and perceived futility of treatment (5 items). The question is rated in a dichotomous format of response (true or false). To exclude the items of mammography in the current study, mammography services are not regularly available and applicable in the study setting and target population. Upon the removal of these items, the resulting version of the scale had 13 items, whose total scores were to fall between 0 and 13 [12]. The higher the scores, the more the cultural beliefs being endorsed that might serve as the hindrance to early recognition and care seeking of breast cancer, and the lower the scores, the lower the number of beliefs.

The translation into Bengali was done using a committee‐based translation strategy so as to have linguistic correctness and cultural appropriateness. First, a questionnaire was translated into Bengali by three bilingual experts on their own. These translators were scholars who had knowledge of public health and were conversant with the Bangladeshi cultures besides being good in the English language. A consensus meeting to address inconsistencies and sharpen language was carried out to review and discuss the translated versions.

The initial Bengali version was then used by the main investigator, oncologist, and another local authority to revise it further to increase the clarity and cultural suitability for adolescent respondents.

### 2.6 Data Analysis

SPSS version 26 was used to analyze the data. Descriptive statistics, including frequencies and percentages, were calculated with respect to categorical variables; means and standard deviation were calculated with respect to categorical variables. Continuous variables had deviations. Certain one‐way analyses (i.e., *t*‐tests and one‐way ANOVA) were calculated to determine the relationship between independent and dependent variables. Multiple linear regression is done to determine the factors associated with cultural belief.

### 2.7 Ethical Considerations

The study protocol was reviewed and approved by the Biosafety, Biosecurity, and Ethical Clearance Committee, BBEC, JU/M 2025/01(175), Jahangirnagar University, Savar, Dhaka‐1342, Bangladesh. Data were collected during school hours in classrooms with the permission of the school's authorities. One day prior to data collection, parent/guardian consent forms were distributed via the class teacher (for minors) for home signature and returned the next day; only students (minors) who returned signed guardian consent were chosen to participate. On the survey day, student assent was obtained prior to questionnaire administration.

## 3 Result

Out of 384 adolescent girls, the majority were between the ages of 13 and 15 years (65.4%), then 16 and 17 years (29.2%), and 17 and 19 years (5.5%). Class 9 was 42.7%, Class 10 was 34.9%, and Class 11–12 was 22.4%. Most of them were single (98.2%) and had the religion of Islam (97.1%). In terms of household income, 62.5% of the respondents gave the monthly income of the family above 30,000 BDT on average, and 37.5% ranked below 30,000. The level of education among fathers was as follows: 5.2% primary education, 26.0% secondary, 39.1% higher secondary, 26.3% graduate level, and 3.4% had no education. In the case of education of mothers, 12.0% were primary educated, 32.3% secondary, 31.0% were higher secondary, 23.2% graduate level, and 1.6% were not educated at all.

Cultural belief scores were significantly higher among participants from lower income households compared to those from higher income households (*F* = 3.50, *p* = 0.001). Father's educational level showed no significant association with cultural belief scores (*F* = 0.723, *p* = 0.576). However, mothers’ educational level was significantly associated with cultural beliefs (*F* = 4.290, *p* = 0.002), with higher mean scores observed among participants whose mothers had primary or higher secondary education compared to those whose mothers were graduates. There are no significant differences in cultural belief scores that were observed across religious groups (*F* = 0.159, *p* = 0.853). Participants with a family history of breast cancer (8.1%) had significantly higher cultural belief scores compared to those without such a history (*t* = 7.982, *p* < 0.001).

Among participants reporting a family history of breast cancer, no significant differences in cultural belief scores were observed on the basis of the relationship with the affected family member (*F* = 0.377, *p* = 0.825). Knowledge about BSE was reported by 29.9% of participants; however, cultural belief scores did not differ significantly between those who had knowledge of BSE and those who did not (*t* = 0.127, *p* = 0.889). Sources of information related to breast health were also examined. No significant differences in cultural belief scores were observed for information obtained from television, radio, books, the internet, or healthcare professionals (*p* > 0.05). However, participants who reported receiving information from family members or friends had significantly lower cultural belief scores compared to those who did not (*t* = −7.861, *p* < 0.001) (Table 2).

**TABLE 2:** Differences in cultural beliefs by general characteristics (*N* = 384).

Characteristics	*N* (%)	Cultural belief (*M* ± SD)	*F*/*t*	*p* values
**Age (years)**				
13–15	251 (65.4)	7.15 (2.03)	1.780	0.170
16–17	112 (29.2)	7.57 (2.07)		
17–19	21 (5.5)	7.46 (2.24)		
**Class of study**				
Class 9 (secondary level)	164 (42.7)	6.93 (1.90)	6.865	**0.001** ^**^
Class 10 (secondary level)	134 (34.9)	7.72 (2.06)		
Class 11 and 12 (intermediate level)	86 (22.4)	7.72 (2.28)		
**Section**				
Science	154 (40.1)	7.61 (2.17)	1.673	0.189
Business study	117 (30.5)	7.15 (1.74)		
Humanities	113 (29.4)	7.32 (2.24)		
**Marital status**				
Married	7 (1.8)	8.14 (0.69)	0.968	0.334
Unmarried	377 (98.2)	7.37 (2.09)		
**Average family monthly income**				
<AMFI (<30,000 BDT)	144 (37.5)	7.86 (1.92)	3.50	**0.001** ^*^
>AMFI (>30,000 BDT)	240 (62.5)	7.10 (2.12)		
**Father's education level**				
Primary level	20 (5.2)	7.35 (1.75)	0.723	0.576
Secondary level	100 (26.0)	7.16 (2.03)		
Higher secondary level	150 (39.1)	7.56 (2.17)		
Graduate	101 (26.3)	7.30 (2.12)		
No education	13 (3.4)	7.76 (1.36)		
**Mother's education level**				
Primary level	46 (12.0)	7.39 (1.91)	4.290	**0.002** ^***^
Secondary level	124 (32.3)	7.49 (2.13)		
Higher secondary level	119 (31.0)	7.83 (1.79)		
Graduate	89 (23.2)	6.66 (2.33)		
No education	6 (1.6)	7.16 (0.98)		
**Religion**				
Islam	373 (97.1)	7.37 (2.09)	0.159	0.853
Hindu	10 (2.6)	7.70 (1.49)		
Others	1 (0.3)	8.00 (0.00)		
**Family history of breast cancer**				
Yes	31 (8.1)	9.35 (1.98)	7.982	**<0.001** ^***^
No	353 (91.9)	7.33 (1.69)		
**Relationship with the patient**				
Mother	7 (1.8)	7.00 (1.41)	0.377	0.825
Sister	7 (1.8)	7.71 (2.49)		
Aunt	12 (3.1)	8.00 (1.65)		
Grandmother	5 (1.3)	7.20 (1.92)		
Unknown	353 (91.9)	7.37 (2.10)		
**Knowledge about BSE**				
Yes	115 (29.9)	7.40 (1.82)	0.127	0.889
No	269 (70.1)	7.37 (2.18)		
**Source of BSE information (multiple answer)**				
Television	23 (6.0)	7.17 (2.12)	−0.508	0.612
Radio	2 (0.5)	5.50 (2.12)	−1.287	0.199
Books	5 (1.3)	7.40 (2.50)	0.013	0.990
Family/Friends	55 (14.3)	5.49 (2.59)	−7.861	**<0.001** ^***^
Internet	102 (26.6)	7.44 (2.07)	0.301	0.764
Healthcare workers/health professionals	11 (2.9)	7.90 (3.20)	0.842	0.400

*Note:* Independent sample *t*‐test was used for variables with two categories, and one‐way ANOVA (*F*‐test) was used for variables with more than two categories. Statistical significance was considered at *p* < 0.05.

Bold values indicate statistically significant associations.

Abbreviations: SD, standard deviation; BSE, breast self‐examination.

* *p* < 0.05.

** *p* < 0.01.

*** *p* < 0.001.

Table 3 presents the distribution of responses to individual items assessing cultural beliefs related to breast cancer among the participants. Beliefs related to breast lump characteristics were common. More than one‐third of respondents believed that a breast lump is not cancer if it is not painful (35.2%) or does not increase in size (41.4%). Additionally, 35.9% believed that frequently touching or pressing a breast lump could cause it to develop into breast cancer.

**TABLE 3:** Participants’ levels of cultural beliefs (*N* = 384).

Domain	Item	Response *n* (%)	
		Right	Wrong
1. Breast lump characteristics	1. If a breast lump is not painful, it is not cancer	135 (35.2)	249 (68.2)
	2. If a breast lump does not get bigger, it is not cancer	159 (41.4)	225 (58.6)
	3. If a breast lump is touched/pressed often, it will turn out to be breast cancer	138 (35.9)	246 (64.1)
2. Self‐help techniques	5. The more you worry about breast cancer, the more likely you will get it	173 (45.1)	211 (54.9)
	6. If you take good care of yourself, you will not get breast cancer	152 (39.6)	232 (60.4)
	13. If you have a breast lump, a “natural” remedy can help to get rid of it	143 (37.2)	241 (62.8)
3. Faith‐based beliefs	7. Faith in God can protect you from breast cancer	100 (26.0)	284 (74.0)
	10. If you pray enough, sometimes breast lumps will disappear	235 (61.2)	149 (38.8)
	14. If a woman has enough faith in God, she will not need treatment for breast cancer	195 (58.0)	189 (49.2)
4. Futility of treatment	11. If breast cancer is cut open in surgery, it will grow faster	193 (50.3)	191 (49.7)
	15. If a woman is poor, she will not get cured from cancer because she will not get the best treatment	132 (34.4)	252 (65.6)
	16. If breast cancer is treated correctly, it can be cured	89 (23.2)	295 (76.8)
	17. It does not really matter if you get treated for breast cancer, because if you get cancer, it will kill you sooner or later	102 (26.6)	282 (73.4)
Total mean score		7.38 ± 2.08	

Regarding self‐help techniques, 45.1% of participants believed that excessive worry about breast cancer increases the likelihood of developing the disease, whereas 39.6% believed that taking good care of oneself can prevent breast cancer. Furthermore, 37.2% believed that natural remedies could eliminate a breast lump, indicating reliance on nonmedical approaches. Faith‐based beliefs were also prevalent among the respondents. Approximately one‐quarter of participants (26.0%) believed that faith in God could protect them from breast cancer. A higher proportion believed that prayer could cause breast lumps to disappear (61.2%), and 58.0% believed that strong faith in God could eliminate the need for medical treatment. Beliefs reflecting the futility of treatment were evident among a substantial proportion of participants. Half of the respondents (50.3%) believed that breast cancer grows faster if surgically operated on. Additionally, 34.4% believed that poverty prevents cures due to limited access to the best treatment. Although 23.2% correctly recognized that breast cancer can be cured if treated appropriately (reverse‐coded item), 26.6% believed that treatment does not alter outcomes because cancer inevitably leads to death.

The overall mean cultural belief score was 7.38 ± 2.08, indicating a moderate level of endorsement of culturally rooted misconceptions related to breast cancer among the participants.

A multiple linear regression (*R* ^2^ = 0.355*, F* = 43.13) analysis was conducted to identify predictors of cultural beliefs about breast cancer among adolescent girls. Class of study was a significant positive predictor of cultural belief scores (*B* = 0.236, SE = 0.113, *β* = 0.089, *t* = 2.097, *p* = 0.037), indicating that students in higher classes were more likely to endorse cultural beliefs related to breast cancer. Family history of breast cancer emerged as a strong negative predictor (*B* = −1.979, SE = 0.181, *β* = −0.466, *t* = −10.930, *p* < 0.001), suggesting that participants with a family history of breast cancer held significantly fewer cultural misconceptions compared to those without such a history. Friends as a source of information was significantly associated with higher cultural belief scores (*B* = 1.358, SE = 0.258, *β* = 0.229, *t* = 5.260, *p* < 0.001), indicating that reliance on peers for information was linked to greater endorsement of cultural beliefs. In contrast, mother's education level (*B* = −0.059, SE = 0.086, *β* = −0.028, *p* = 0.497) and average monthly family income (*B* = −0.277, SE = 0.182, *β* = −0.065, *p* = 0.128) were not statistically significant predictors of cultural belief scores (Table 4).

**TABLE 4:** Multiple linear regression analysis for factors associated with cultural beliefs.

Predictor	*B*	SE	*β*	*t*	*p*	95% CI
Class of study	0.236	0.113	0.089	2.097	**0.037** ^*^	[0.015, 0.458]
Mother's education level	−0.059	0.086	−0.028	−0.680	0.497	[−0.228, 0.111]
Family history of breast cancer	−1.979	0.181	−0.466	−10.930	**<0 001** ^**^	[−2.335, −1.623]
Friends	1.358	0.258	0.229	5.260	**<0.001** ^**^	[0.851, 1.866]
Average monthly income	−0.277	0.182	−0.065	−1.525	0.128	[−0.635, 0.080]

*Note: B* = unstandardized coefficient; *β* = standardized coefficient. Results are based on multiple linear regression analysis.

Bold values indicate statistically significant associations.

Abbreviations: CI, 95% confidence interval; SE, standard error.

* *p* < 0.05.

** *p* < 0.001.

## 4 Discussion

This study provides an insight into knowledge, perception, and predictors of cultural belief related to breast cancer among female high school students in semi‐urban areas of Bangladesh.

In general, the participants in this study had a total cultural belief score of (7.38 out of 13) moderate amount of culturally related misconceptions regarding breast cancer. This is higher than the women in rural Vietnam and Turkey, who had overall a low cultural belief score of 3.4 and 3.24 out of 13, respectively [12, 23]. This variation could be due to several reasons. First, the Vietnamese participants were adult women aged 20–64 years who had been exposed to the organized national cancer control program in Vietnam and community‐based breast cancer screening programs over time [12], whereas the adolescent participants in the present study had not had a similar exposure to structured breast‐health education in the school curriculum. Second, the cultural and religious meaning of illness in Bangladesh, a predominantly Muslim, socially South Asian context, is different than in the Vietnamese and Turkish contexts. In South Asia, breast cancer has been strongly associated with well‐established beliefs about female reproductive issues that promote taboos regarding modesty and secretiveness, thereby actively discouraging public discussion of breast cancer even among family members [5, 24]. This stigma can perpetuate a larger pool of culturally embedded myths about these relationships among Bangladeshi teenagers who face the risks of these relationships with less formal education or experience to question them. Third, developmental and life‐stage differences between the two samples likely play a role. The Turkish and Vietnamese participants were adult women aged 20+, many of whom had already been pregnant, undergone routine health checkups, or interacted with healthcare providers, who encounter natural opportunities to correct inaccurate beliefs through direct clinical guidance [12, 23]. Adolescent participants in the present study, by contrast, have had little personal contact with healthcare providers regarding breast cancer and instead form their beliefs primarily through informal channels, such as peers, family conversation, and unregulated online content [18]. Without this corrective clinical contact, misconceptions acquired informally during adolescence may persist unchallenged.

Our study found that cultural belief scores increased significantly with higher class of study, indicating that students in advanced grades held stronger culturally rooted beliefs about breast cancer. This finding is partially inconsistent with studies conducted among adult women and university students, where higher educational attainment is associated with greater health and science‐related capabilities [24, 25]. However, similar patterns have been observed in recent study in China; cancer literacy strongly tracks education level, yet even better educated groups have the weakest literacy in prevention and risk factors [8]. This discrepancy may be explained by the absence of structured breast‐health education in secondary and higher secondary school curriculum in Bangladesh [26]. As adolescents advance academically, both the internet and parents, with friends, become especially important for sensitive topics (e.g., sexuality) [18]. In the absence of accurate guidance, these exposures may reinforce cultural myths rather than correct them. Thus, unlike higher education settings, secondary schooling alone may be insufficient to counter deeply ingrained cultural beliefs during adolescence.

Our study found that adolescents with a family history of breast cancer exhibited significantly lower cultural belief scores. Similar findings have been reported in studies from the Middle East and North Africa, knowing that a family member or friend with breast cancer had a stronger impact on women's self‐care and screening than education or employment [27].

One of the most important findings is that adolescents who recommend friends as a source of information had significantly higher cultural belief scores. This finding is consistent with recent large reviews and meta‐analyses, cross‐domain effects on adolescent behavior, and attitudes [19]. However, it contrasts with studies among older youth and university students where peer education showed significant gains in breast cancer knowledge, health responsibility, health beliefs, and BSE practices [28]. The divergence likely reflects differences in informational quality rather than the influence mechanism itself. Among younger adolescents in semi‐urban Bangladesh, peer networks may function as echo chambers for misinformation, rumors, and culturally transmitted myths, particularly in environments where adult guidance and formal health education are limited. This contrasts with university environments, where peer discussions are more likely to be informed by academic exposure and access to credible information sources.

Contrary to several studies conducted among adult women, this study found no significant association between cultural belief scores and household income or mother's education level in the multivariable model. Previous research has often reported higher education, income, and health insurance jointly improve breast cancer knowledge, attitudes, and preventive behaviors, whereas low SES and cultural beliefs sustain misinformation and delayed diagnosis [29]. This null result is also different from the study of Kim et al. who found a significantly higher cultural belief scores among women who had lower household income [12]. This gap could be because adolescents are more likely to report on how their parents earn income than on their own and may not be as vulnerable to the economic constraints that affect their ability to access health information as those that affect adult women's decisions about their own health information; family income therefore may be a less accurate indicator of an adolescent's actual exposure to health information than it is for adult women.

The high prevalence of faith‐based beliefs observed in this study is consistent with findings from other South Asian and Muslim‐majority contexts, where religious interpretations often coexist with biomedical explanations of disease [11]. Beliefs that prayer alone can eliminate illness or that cancer is inevitably fatal have been widely documented and are associated with delayed care‐seeking in adulthood.

This study has several limitations that should be considered when interpreting the findings. First, cross‐sectional design prevents causal inferences regarding the relationships between sociodemographic factors and cultural beliefs about breast cancer. Second, the study was conducted among female students from semi‐urban schools in a limited geographic area that may restrict the generalizability of the results to adolescents in rural or urban settings. Third, data collection relied on a single self‐administered questionnaire without triangulation from qualitative or observational methods, and the test–retest reliability of the translated 13‐item scale was not formally assessed in this sample; reliance on self‐reported data may also have introduced reporting or social desirability bias, particularly given the sensitive nature of breast‐health topics in the local cultural context. Fourth, the sample size calculation using Yamane's formula did not incorporate a design–effect adjustment for the stratified sampling procedure, which may understate the precision achieved. Fifth, several sociodemographic variables examined (e.g., religion, marital status) showed limited variability within this population, reducing statistical power to detect associations for those factors. Finally, the absence of qualitative data limits a deeper exploration of the underlying reasons and contextual meanings behind the observed cultural beliefs.

## 5 Conclusion

This study shows that many adolescent girls in semi‐urban Bangladesh hold cultural beliefs and misconceptions about breast cancer that may influence how they understand the disease and respond to it in the future. Such beliefs may be influenced by levels of school, family exposure to breast cancer, and friends. Due to the importance of teens as a period during which long‐term health attitudes are established, it is necessary to inform them about misconceptions at an early age. Elementary and culturally sensitive interventions could be undertaken in schools to make the research understandable and lower the pernicious beliefs through the incorporation of peers, families, and trusted community members.

## Author Contributions

Conceptualization: Ryadul Alam, Sadia Akter, and Md. Tajuddin Sikder. Data collection and data entry: Sadia Akter, Tanaa Mohammad, Rowshanara Rini, Mst. Mariyam, Afrana Akter Priti, Aishwaria Mitra Ayshee, Md. Shahriar Hossen Sayem, and Afsana Mohammad Mimi. Formal analysis: Ryadul Alam, Sadia Akter, and Zubaida Iftekhar. Investigation: Md. Habibullah Talukder and Md. Tajuddin Sikder. Methodology: Ryadul Alam, Md. Tajuddin Sikder, and Zubaida Iftekhar. Supervision: Md. Habibullah Talukder and Md. Tajuddin Sikder. Validation: Md. Habibullah Talukder. Writing – original draft: Ryadul Alam, Sadia Akter, Tanaa Mohammad, Rowshanara Rini, Mst. Mariyam, Afrana Akter Priti, Aishwaria Mitra Ayshee, and Md. Shahriar Hossen Sayem. Writing – review and editing: Md. Habibullah Talukder and Zubaida Iftekhar.

## Funding

The authors have nothing to report.

## Disclosure

A preprint of this manuscript was previously posted on Research Square prior to submission to *Public Health Challenges*: Alam MR, et al. “Cultural Beliefs and Perceptions of Breast Cancer Among Adolescent Girls in Semi‐Urban Bangladesh: A School‐Based Cross‐Sectional Study.” Research Square [Preprint]. DOI: https://doi.org/10.21203/rs.3.rs‐8628237/v1.

## Ethics Statement

The study was approved by the Biosafety, Biosecurity & Ethical Committee, Faculty of Biological Sciences, Jahangirnagar University (Approval No. BBEC, JU/M 2025/01(175)).

## Consent

Written informed consent for publication of anonymized data was obtained from all participants and their legal guardians.

## Conflicts of Interest

The authors declare no conflicts of interest.

## Data Availability

The datasets supporting the conclusions of this article are available from the corresponding author upon reasonable request.
